# *Coxiella burnetii* Seroprevalence and Risk for Humans on Dairy Cattle Farms, the Netherlands, 2010–2011

**DOI:** 10.3201/eid2003.131111

**Published:** 2014-03

**Authors:** B. Schimmer, N. Schotten, E. van Engelen, J.L.A. Hautvast, P.M. Schneeberger, Y.T.H.P. van Duijnhoven

**Affiliations:** National Institute for Public Health and the Environment (RIVM), Bilthoven, the Netherlands (B. Schimmer, N. Schotten, Y.T.H.P. van Duynhoven);; Animal Health Service, Deventer, the Netherlands (E. van Engelen);; Radboud University Nijmegen Medical Centre, Nijmegen, the Netherlands (J.L.A. Hautvast);; Jeroen Bosch Hospital, ‘s-Hertogenbosch, the Netherlands (P.M. Schneeberger)

**Keywords:** Coxiella burnetii, Q fever, seroepidemiology, dairy cattle, farm residents, farm workers, farmers, risk factors, bacteria, zoonoses, the Netherlands, dairy farms, dairy cattle farms, seroprevalence, BTM, bulk tank milk

## Abstract

Q fever, caused by *Coxiella burnetii*, is a recognized occupational infection in persons who have regular contact with ruminants. We determined *C. burnetii* seroprevalence in residents living or working on dairy cattle farms with >50 adult cows and identified risk factors for seropositivity. Serum samples from farm residents, including employees, were tested for *C. burnetii* IgG and IgM; seroprevalence was 72.1% overall and 87.2%, 54.5%, and 44.2% among farmers, spouses, and children, respectively. Risk factors included farm location in southern region, larger herd size, farm employment, birds in stable, contact with pigs, and indirect contact with rats or mice. Protective factors included automatic milking of cows and fully compliant use of gloves during and around calving. We recommend strengthening general biosecurity measures, such as consistent use of personal protective equipment (e.g., boots, clothing, gloves) by farm staff and avoidance of birds and vermin in stables.

Q fever is an occupational zoonosis caused by *Coxiella burnetii*, a gram-negative bacterium (*1*). Ruminant farmers, laboratory workers, dairy workers, and veterinarians are at particular risk for infection. Humans usually acquire Q fever by inhalation of *C. burnetii* aerosolized from contaminated materials originating from infected animals. The primary animal reservoirs responsible for human infections are cattle, sheep, and goats, which can shed *C. burnetii* in urine, feces, milk, and birth products. Before 2007, the seroprevalence of *C. burnetii* antibodies within the general population of the Netherlands was 2.4%; keeping ruminants and increasing age were risk factors for seropositivity (*2*). During 2007–2009, Q fever was a major public health problem in the Netherlands; >4,000 human cases were reported (*3*). Large-scale interventions primarily targeting small ruminants were used to control the epidemic. In 2008, mandatory vaccination was conducted in a defined cluster area and later nationwide. In 2009–2010, a program was implemented to cull pregnant dairy goats and sheep on farms with *C. burnetii*–positive animals identified through a national bulk tank milk (BTM) screening (*4*). Since then, the incidence of acute Q fever cases has diminished substantially (*5*), but chronic cases still occur (*6*). No epidemiologic associations between Q fever cases in humans and dairy cattle were identified during this epidemic, nor have any been described in other Q fever outbreaks (*7*). Nevertheless, recent reports indicate that *C. burnetii* is widespread among Dutch dairy cattle herds (prevalence 78.6% [ELISA] or 56.6% [PCR] in BTM samples) (*8*). In 2008, seroprevalence was 16.0% in lactating cows and 1.0% in young animals (*8*).

*C. burnetii* seroprevalence estimates for dairy cattle farm residents in the Netherlands are outdated, and risk factors associated with seropositivity are seldom studied. This lack of data inhibits accurate assessment of the public health risk. To inform control measures and provide advice for persons living/working on a dairy cattle farm (DCF), we conducted a cross-sectional study to investigate the seroprevalence of *C. burnetii* antibodies in DCF residents/workers and identified participant-based and farm-based risk factors for seropositivity. The study was approved by the Medical Ethics Committee of the University Medical Centre Utrecht (no. 09–189/K).

## Methods

A total of 3,000 DCFs housing >50 adult dairy cows were randomly selected for possible participation in the study from a national database maintained by the Animal Health Service. In September and November 2010, information and recruitment materials were sent to 1,000 and 2,000 farms, respectively. Farms were enrolled in the study after returning a completed informed-consent form. After 4 weeks, nonresponding farms from the first mailing received a written reminder. Nonresponding farms from the second mailing did not receive a reminder because the goal of enrolling 296 farms had been reached; this number was determined on the basis of power calculations assuming 50.0% prevalence and 5.5% precision. We contacted enrolled farms by telephone to confirm participation and determine the number of participants. Dairy cattle farmers and up to 2 family members or farm employees >12 years of age were eligible for participation in the study. Participants completed a questionnaire about personal characteristics (e.g., age, medical history, farm-related activities, contact with livestock and companion animals, consumption of unpasteurized dairy products, and use of personal protective equipment [PPE]) and provided a serum sample (collected by a laboratory assistant during a home visit). The farm owner or manager completed a questionnaire about herd size, cattle housing, presence of other livestock and companion animals, farm facilities, animal health, and hygiene measures. Participating farms were requested to provide one BTM sample for testing by ELISA and PCR, as described (*8*).

### Serology

We used an immunofluorescence assay (IFA) (Focus Diagnostics, Cypress, CA, USA) to test serum samples for *C. burnetii* phase I and II IgM and IgG. All samples were screened at an initial dilution of 1:32; those with negative results were considered negative. Positive samples were further classified as indicative of relatively recent infections (IgM phase II titer >32) or past infections (IgG phase II titer >32 and IgM phase II titer <32). Samples with all other outcomes were considered negative. The term relatively recent was chosen because phase II IgM is commonly found up to 1 year after infection in acute Q fever cases, but it may persist up to 3 years (*9*). Phase I and II IgG end point titers were determined for all seropositive persons. In agreement with chronic Q fever diagnostic criteria used in the Netherlands (*10*), phase I IgG titers ≥1,024 in samples in the past infection group were considered indicative of possible chronic infection.

### Data Analysis

Participating and nonparticipating farms were compared with respect to herd size; distance to nearest *C. burnetii*–positive BTM small-ruminant farm; goat, sheep, and cattle density; location by province and region; and degree of urbanization. We used the Mann-Whitney U test to determine differences in continuous variables and the χ^2^ test to analyze categorical variables. We performed univariate logistic regression analyses to determine the main factors associated with *C. burnetii* seropositivity among participants (p<0.20, likelihood ratio test). Potential farm-based risk factors were analyzed by univariate multilevel analyses; a unique farm identifier was used as the cluster variable. Distributions of continuous variables were studied, and variables not linearly related to the outcome variable were categorized on the basis of biological arguments (e.g., nearest *C. burnetii*–positive BTM small-ruminant farm) or, if those were lacking, on medians (e.g., goat density within 5-km radius). Participant age was always kept in the model because of its frequent relation with seropositivity. Variables with <10.0% of participants in a risk category were excluded from further analysis. If several variables were found interrelated in the univariate analysis, only the most informative and relevant variable was selected for inclusion.

Risk factors determined to be significant (p<0.20) in univariate analyses of the participant-based and farm-based data were incorporated into multivariate logistic regression and multivariate multilevel analyses, respectively. Stratified multivariate analyses for participant risk factors were performed separately for farmers and for the remaining group. Starting with a full model, manual backward elimination was performed; all variables meeting the 10.0% significance level in the likelihood ratio test were kept in the final model. Two-way interactions between biologically plausible variables in the multivariate model were investigated. Last, variables included in the final multivariate model for participant-based factors and those included in the multilevel model for farm-based factors were combined in a multivariate multilevel analysis to identify the independent risk determinants for seropositivity. The final model fit was assessed by the quasi-likelihood under the independence model criterion goodness-of -fit statistic for generalized estimation equation models. SAS version 9.2 (SAS Institute, Cary, NC, USA) was used for all analyses.

## Results

### Nonresponse Analysis

Of the 3,000 invited farms, 311 provided a BTM sample, and 755 persons from 309 (10.3%) farms participated in this study by providing a serum sample. A farm-based questionnaire was available for 736 (97.5%) persons from 301 farms, and a participant-based questionnaire was completed by 729 (96.6%) persons from 308 farms. Compared with nonparticipating farms, participating farms were a median of 1.5 km closer to small ruminant farms with *C. burnetii*–positive BTM samples (Table 1). In addition, the density of sheep within a 5-km radius of participating farms was higher than that for nonparticipating farms; however, the absolute difference was very small (3 sheep/km^2^).

**Table 1:** Nonresponse analyses of farms in a study of *Coxiella burnetii* seroprevalence and risk for seropositivity in humans on dairy cattle farms, the Netherlands, September 2010–March 2011

Variable	Farms		p value
	Participating, n = 311	Nonparticipating, n = 2,685	
Categorical, no. (%)			
Farm located inside vaccination area	83 (26.4)	590 (21.9)	0.08
Farm region*			0.36
North	80 (25.4)	781 (29.1)	
East	104 (33.7)	911 (33.9)	
West	57 (18.7)	494 (18.3)	
South	70 (22.2)	503 (18.7)	
Degree of urbanization of the farm municipality			0.77
Moderately, strongly, or extremely (>1,000 addresses/km^2^)	1 (0.3)	17 (0.6)	
Hardly (500–1,000 addresses/km^2^)	10 (3.2)	94 (3.5)	
Not (<500 addresses/km^2^)	300 (96.5)	2,574 (95.9)	
Numerical, median no.			
No. cows in 2008			
<1	35	35	0.44
1–2	26	26	0.65
>2	85	86	0.16
Nearest bulk tank milk positive small-ruminant farm (meters)	9,793	11,301	0.01
Goat density (animals/km^2^)†			
Within 5-km radius	9.2	6.7	0.27
Within 10-km radius	9.3	9.2	0.26
Sheep density (animals/km^2^)†			
Within 5-km radius	30	33	0.04
Within 10-km radius	34	35	0.11
Cattle density (animals/km^2^) within 5-km radius†			
Including own animals	178	181	0.29
Excluding own animals	175	179	0.27
Cattle density (animals/km^2^) within 10-km radius†			
Including own animals	170	170	0.99
Excluding own animals	169	169	0.91

*North represents Groningen, Friesland, and Drenthe Provinces; East represents Gelderland, Overijssel, and Flevoland Provinces; West represents Noord–Holland, Zuid–Holland, Utrecht, and Zeeland Provinces; and South represents Limburg and Noord–Brabant Provinces. †Corrected for area in the Netherlands.

### Seroprevalence

Overall *C. burnetii* seroprevalence was 72.1% (95% CI 68.8%–75.3%), and seroprevalence among farmers, spouses, and children (12–17 years of age) was 87.2%, 54.5%, and 44.2%, respectively (Table 2). Seroprevalence was univariately significantly higher among male participants, farmers, and participants >35 years of age (Table 3, Appendix, wwwnc.cdc.gov/EID/article/20/3/13-1111-T3.htm). The median duration of farm residence was 28 years (range 0–56). IgG phase II end titers were known for 534 (98.9%) of 540 *C. burnetii* IgG phase II–seropositive participants: 32 (n =166), 64 (n = 92), 128 (n = 119), 256 (n = 106), 512 (n = 39), 1,024 (n = 10), 2,048 (n = 1), and 4,096 (n = 1). IgG phase I end titers were known for 283 (97.6%) of the 290 IgG phase I–seropositive participants: 32 (n = 105), 64 (n = 73), 128 (n = 61), 256 (n = 32), 512 (n = 10), 1,024 (n = 1), and 2,048 (n = 1). These last 2 participants, with phase I titers of 1,024 and 2,048, respectively, had lower IgG phase II titers (512 and 1,024, respectively), and according to chronic Q fever diagnostic criteria used in the Netherlands (*10*), these participants met the conditions for possible chronic Q fever infection. We could not confirm that these truly were chronic Q fever cases because clinical information (e.g., presence of vascular infection, endocardial involvement, or other clinical risk factors) was lacking.

**Table 2:** Participant characteristics and *Coxiella burnetii* seroprevalence among dairy cattle farm residents, the Netherlands, September 2010– March 2011

Participant characteristic	Total no. residents/no. positive (%)	95% CI
All participants	755/544 (72.1)	68.8–75.3
Sex		
M	431/368 (85.4)	82.0–88.7
F	323/176 (54.5)	49.0–59.9
Age, y		
<35	169/107 (63.3)	56.0–70.7
35–44	176/131 (74.4)	67.9–80.9
45–54	252/185 (73.4)	67.9–78.9
>55	132/106 (80.3)	73.4–87.2
Role		
Farmer	361/315 (87.2)	83.8–90.7
Spouse	222/121 (54.5)	47.9–61.1
Child <18 y	52/23 (44.2)	30.3–58.2
Child >18 y	54/40 (74.1)	62.0–86.1
Other*	40/30 (75.0)	61.0–89.0

*Represents other family members and employees.

**Table 3,:** Appendix. Univariate logistic model of participant-based characteristics associated with *Coxiella burnetii* seropositivity among dairy cattle farm residents, the Netherlands, September 2010– March 2011*

Variable	No. residents (% positive)	OR (95% CI)
Sex		
M	431 (85.4)	4.9 (3.5–6.9)
F	323 (54.5)	Reference
Age, y†		
<35	169 (63.3)	Reference
35–44	176 (74.4)	1.7 (1.1–2.7)
45–54	252 (73.4)	1.6 (1.1–2.4)
>55	132 (80.3)	2.4 (1.4–4.0)
Role		
Farmer	361 (87.3)	5.7 (3.8–8.6)
Spouse	222 (54.5)	Reference
Child <18 y	52 (44.2)	0.7 (0.4–1.2)
Child >18 y	54 (74.1)	2.4 (1.2–4.6)
Other‡	40 (75.0)	2.5 (1.2–5.4)
Presence in stable		
Every day	584 (78.3)	8.4 (2.1–32.9)
Every week	117 (51.3)	2.5 (0.6–10.0)
Every month	18 (50.0)	2.3 (0.5–12.0)
<1 time/month	10 (30.0)	Reference
Work on the farm†		
No	42 (31.0)	Reference
Part time (1–39 h/wk)	333 (60.7)	3.3 (1.7–6.6)
Full time (>40 h/wk)	354 (88.7)	17.5 (8.4–36.4)
Care for calves		
Yes	501 (78.4)	2.5 (1.8–3.5)
No	228 (59.6)	Reference
Milk cows		
Yes	442 (84.4)	4.5 (3.2–6.4)
No	287 (54.4)	Reference
Feed cattle		
Yes	515 (81.6)	4.3 (3.0–6.0)
No	214 (50.9)	Reference
Transport cattle		
Yes	414 (85.0)	4.4 (3.1–6.3)
No	315 (56.2)	Reference
Provide health care for cattle		
Yes	440 (83.0)	3.7 (2.7–5.3)
No	288 (56.6)	Reference
Provide birth care for cattle		
Yes	516 (80.8)	3.8 (2.7–5.4)
No	212 (52.4)	Reference
Remove manure		
Yes	478 (83.3)	4.6 (3.2–6.4)
No	251 (52.2)	Reference
Spread manure		
Yes	254 (86.6)	3.5 (2.3–5.2)
No	474 (65.2)	Reference
Clean stables		
Yes	505 (80.2)	3.3 (2.3–4.6)
No	222 (55.4)	Reference
No. of tasks (0–9) performed on farm†§	725 (72.7)	1.3 (1.2–1.4)
Wear overalls†		
Almost every time/regularly	584 (76.7)	2.5 (1.7–3.6)
Sometimes/never	142 (57.0)	Reference
Wear boots†		
Almost every time/regularly	646 (74.6)	2.1 (1.3–3.4)
Sometimes/never	81 (58.0)	Reference
Use gloves during cattle birth care†		
Fully compliant	61 (70.5)	0.5 (0.3–0.9)
Partly or noncompliant	456 (82.2)	Reference
No birth care	211 (52.4)	0.2 (0.2–0.3)
Use disinfectant during cattle birth care		
Fully compliant	141 (82.3)	1.1 (0.7–1.9)
Partly or noncompliant	374 (80.5)	Reference
No birth care	212 (53.3)	0.3 (0.2–0.4)
Have contact with pigs at own or other farm†¶		
Yes	83 (85.5)	2.4 (1.3–4.6)
No	646 (70.9)	Reference
Have contact with sheep at own or other farm†¶		
Yes	232 (75.9)	1.3 (0.9–1.8)
No	497 (71.0)	Reference
Have contact with poultry at own farm†¶		
Yes	177 (67.8)	0.8 (0.5–1.1)
No	578 (73.4)	Reference
Have indirect contact with rats/mice at own farm†#		
Yes	213 (82.6)	2.2 (1.5–3.3)
No	516 (68.4)	Reference
Have contact with dogs at own farm**		
Yes	538 (71.0)	0.7 (0.5–1.1)
No	190 (76.8)	Reference
Have contact with dogs at other farm†**		
Yes	273 (75.8)	1.3 (0.9–1.8)
No	456 (70.6)	Reference
Have contact with cows at other farm†**		
Yes	195 (82.6)	2.2(1.4–3.3)
No	532 (68.8)	Reference
Have indirect contact with cows at other farm#		
Yes	326 (77.6)	1.6 (1.1–2.3)
No	400 (68.3)	Reference
Have contact with compost†		
Yes	139 (66.2)	0.7 (0.5–1.0)
No	590 (74.1)	Reference
Have contact with hay†		
Daily (almost)	505 (80.2)	3.3 (2.3–4.6)
Weekly or less often	222 (55.4)	Reference
Have contact with cattle food†		
Daily (almost)	530 (80.9)	4.2 (3.0–6.0)
Weekly or less often	199 (50.3)	Reference
Have contact with raw milk†		
Daily (almost)	507 (78.9)	2.7 (1.9–3.8)
Weekly or less often	221 (57.9)	Reference
Have contact with sheep or goat manure†		
Yes	96 (80.2)	1.6 (1.0–2.8)
No	632 (71.4)	Reference
Have contact with cattle manure†		
Daily (almost)	477 (83.0)	4.4 (3.1–6.2)
Weekly or less often	252 (52.8)	Reference
Have contact with live–born animals		
Yes	626 (76.7)	3.7 (2.4–5.7)
No	102 (47.1)	Reference
Have contact with stillborn animals		
Yes	304 (83.9)	2.9 (2.0–4.1)
No	424 (64.5)	Reference
Have contact with placenta material or amniotic fluid†		
Yes	425 (84.2)	4.2 (2.9–5.9)
No	304 (56.3)	Reference
Consume Brie cheese†		
Yes	166 (63.9)	0.6 (0.4–0.9)
No	563 (75.1)	Reference
Lived on a farm with animals during childhood†		
Yes	604 (76.4)	2.8 (1. 9–4.2)
No	125 (53.6)	Reference

*Analysis was performed to assess the main participant-based characteristics associated with positivity (p<0.20 in likelihood ratio test). OR, odds ratio. †Variables included in later multivariate analysis before manual backward elimination. ‡Represents other family members and employees. §No. tasks (i.e., caring for calves, milking, feeding cattle, transport of cattle, general cattle health, assistance during calving, removing manure, spreading manure and cleaning the stables) performed on the farm by 1 person (0–9 tasks). Risk increases per task. ¶See animals at <5 meters or touch animals (contact). #See animals at <5 meters (indirect contact). **Touch animals (direct contact).

Nine (1.2%) participants from 8 farms were classified as having a relatively recent infection (IgM phase II titer range 32–256). All 8 farms were within 2.5–21.2 km of the nearest *C. burnetii*–positive BTM small-ruminant farm, and 4 of the 8 were within 3 km.

Four participants reported having had Q fever diagnosed by a physician during 2008–2010. On the basis of serum samples obtained at study entry, 3 of these participants had a serologic profile indicating past infection. These 3 participants lived in the southern or eastern region of the Netherlands on farms within a 3-km radius of the nearest small-ruminant farm with *C. burnetii*–positive BTM samples. The fourth participant had no serologic evidence of a past infection and lived 14 km from the nearest small-ruminant farm with *C. burnetii*–positive BTM samples.

### Univariate Analyses at Participant and Farm Levels

Risk factors for seropositivity for farmers/workers and residents included age >35 years; farm employment; directly performing cattle-related tasks; contact with cattle, pigs, hay, cattle food, raw milk, manure, or cattle birth products; presence of rats or mice on the farm; and growing up on a farm (Table 3, Appendix). Protective factors included poultry and compost contact and fully compliant use of gloves during and around calving. Farm-based risk factors included a larger herd size, farm location in the southern region, an annual peak in calving, having beef cattle on the farm, and the presence of birds in the stable. Protective factors included automatic milking, having pet cats or rabbits, and having farm clothes and boots available for professional visitors (e.g., veterinarians and feed specialists) (Table 4). No relationship was found between PCR or ELISA status on the basis of BTM samples and participant seropositivity.

**Table 4:** Univariate logistic model of farm-based characteristics associated with *Coxiella burnetii* positivity among dairy cattle farm residents, the Netherlands, September 2010–March 2011*

Variable	No. residents total (% positive)	OR (95% CI)
No. cows on farm in 2008†‡	755 (72.1)	1.0 (1.0–1.0)
Nearest bulk tank milk positive small-ruminant farm†		
<8 km	331 (75.8)	1.4 (1.0–1.9)
>8 km	424 (69.1)	Reference
Municipal cattle density, including beef calves§	755 (72.1)	1.0 (1.0–1.0)
Farm location		
Inside small-ruminant vaccination area	202 (78.2)	1.6 (1.0–2.3)
Outside small-ruminant vaccination area	553 (69.8)	Reference
Farm region†		
South	170 (80.6)	1.8 (1.2–2.7)
Other	585 (69.6)	Reference
Beef cattle on the farm†		
Yes	79 (82.3)	1.9 (1.1–3.4)
No	652 (70.7)	Reference
Annual peak in calving		
Yes	135 (76.3)	1.3 (0.9–2.0)
No	601 (71.1)	Reference
Automatic milking†		
Yes	154 (65.6)	0.7 (0.5–1.0)
No	580 (73.8)	Reference
Use of bedding in stables		
Yes	717 (72.4)	1.9 (1.2–2.9)
No	19 (57.9)	Reference
Pet cat		
Yes	444 (69.1)	0.6 (0. 5–0.9)
No	285 (77.9)	Reference
Pet rabbit		
Yes	202 (64.4)	0.6 (0.4–0.8)
No	527 (75.7)	Reference
Birds in stable†		
Yes	90 (82.2)	1.9 (1.0–3.6)
No	644 (70.5)	Reference
Use of by-product feedstuffs†		
Yes	229 (77.3)	1.5 (1.0–2.1)
No	507 (69.6)	Reference
No. cows that calved in 2009‡	720 (71.8)	1.0 (1.0–1.0)
No. live-born calves		
<78	335 (69.0)	Reference
>78	344 (74.1)	1.3 (0.9–1.8)
No. twin calves		
1–2	272 (69.9)	Reference
>3	313 (76.4)	1.4 (1.0–2.0)
Type of farm management†		
Closed herd	515 (73.4)	Reference
Purchase of cattle	213 (68.1)	0.8 (0.6–1.1)
No. cattle purchase addresses in 2007†		
0 or 1	649 (72.7)	Reference
>2	76 (64.5)	0.7 (0.4–1.0)
Farm boots and work clothes available for professional visitors		
Yes	662 (71.3)	0.7 (0.4–1.1)
No	74 (78.4)	Reference
Work clothes available for own personnel		
Yes	556 (73.6)	1.4 (1.0–1.9)
No	180 (67.2)	Reference

*The analysis included the primary farm-based factors associated with positivity (p<0.20 in likelihood ratio test). OR, odds ratio. †Variable included in later multivariate analysis before manual backward elimination. ‡Risk increases per cow. §Risk decreases per cow.

### Multivariate and Multilevel Analyses

Of the 21 variables considered in the multivariate participant model, 8 were independently associated with seropositivity: age >55 years; working on the farm; fully compliant use of gloves during cattle birth care; contact with pigs, cattle at other farms, poultry, or compost; and indirect contact with rats or mice (Table 5). Interaction terms did not improve the model.

**Table 5:** Multivariate logistic regression analysis of participant-based characteristics associated with *Coxiella burnetii* positivity among dairy cattle farm residents, the Netherlands, September 2010–March 2011*

Association with positivity, characteristic	OR (95% CI)
Positive association	
Age, y	
<35	Reference
35–44	1.4 (0.8–2.3)
45–54	1.0 (0.6–1.6)
>55	1.9 (1.0–3.5)
Work on farm	
No	Reference
Part time (1–39 h/wk)	2.4 (1.1–5.2)
Full time (>40 h/wk)	10.4 (4.2–25.7)
Contact with pigs at own or other farm	
Yes	2.6 (1.2–5.4)
No	Reference
Contact with cows at other farm	
Yes	1.6 (1.0–2.6)
No	Reference
Indirect contact with rats/mice at own farm	
Yes	1.5 (1.0–2.4)
No	Reference
Negative association	
Use of gloves during cattle birth care	
Fully compliant	0.4 (0.2–0.8)
Partly or noncompliant	Reference
No birth care	0.7 (0.4–1.1)
Contact with poultry at own farm	
Yes	0.6 (0.4–0.9)
No	Reference
Contact with compost	
Yes	0.6 (0.3–0.9)
No	Reference

*The analysis included the primary participant-based characteristics associated with positivity (p<0.10 in likelihood ratio test). The number of observations was 712. Model fit was assessed by use of the Hosmer–Lemeshow goodness-of-fit test (p = 0.91). OR, odds ratio.

Of the 9 variables considered in the multilevel farm model, 6 were independently associated with seropositivity; larger herd size, farm location in the southern region, beef cattle on the farm, use of food concentrate, and presence of birds in the stable were risk factors, and automatic milking was a protective factor (Table 6). In the combined multilevel analysis, the 12 significant factors from the multivariate participant and multilevel farm models, in addition to age, were combined in 1 model. The nonstratified model had a clearly better fit than the stratified model for farmers. Farm location within 8 km of the nearest *C. burnetii*–positive BTM small-ruminant farm (odds ratio 2.3, 95% CI 1.2%–2.5%) was a risk factor in the final stratified multilevel model among farmers and was therefore included in the combined multilevel analysis. In the final overall model, independent risk factors were age >55 years, farm employment, pig contact, larger herd size, farm location in the southern region, beef cattle on the farm, cattle contact at other farms, and presence of birds in the stable. Indirect contact with rats or mice was borderline significant (Table 7). Protective factors were contact with poultry or compost, use of automatic milking, and fully compliant use of gloves during birth care. We ran an additional model by adding a protective variable (farm clothes and boots available for professional visitors), as described in Table 5, in the farm-based and combined multilevel models. Doing so resulted in a final model with the same factors as shown in Table 7, except that automatic milking was replaced by another protective factor (farm clothes and boots available for professional visitors) and 2 borderline significant risk factors (distance to the nearest *C. burnetii*–positive BTM small-ruminant farm and use of by-product feedstuffs) (data not shown).

**Table 6:** Multilevel analysis of farm-based characteristics as independent factors associated with *Coxiella burnetii* positivity among dairy cattle farm residents, the Netherlands, September 2010–March 2011*

Variable	OR (95% CI)
No. cows on farm in 2008†	1.0 (1.0–1.0)
Farm region	
South	1.8 (1.2–2.8)
Other	Reference
Beef cattle on farm	
Yes	1.7 (1.0–2.8)
No	Reference
Automatic milking	
Yes	0.7 (0.4–1.0)
No	Reference
Birds in stable	
Yes	2.0 (1.1–3.8)
No	Reference
Use of by-product feedstuffs	
Yes	1.4 (1.0–2.0)
No	Reference

*The analysis included the primary farm-based characteristics associated with positivity (p<0.10 in likelihood ratio test). The number of observations was 716; the number of levels used was 309 (quasi-likelihood under the independence model criterion 832.88). OR, odds ratio. †Risk increased per cow.

**Table 7:** Combined multilevel analysis of participant- and farm-based characteristics associated with *Coxiella burnetii* seropositivity in dairy cattle farm residents, the Netherlands, September 2010–March 2011*

Variable	OR (95% CI)
Age, y	
<35	Reference
35–44	1.3 (0.8–2.3)
45–54	1.2 (0.7–2.0)
>55	1.9 (1.1–3.5)
Work on farm	
No	Reference
Part time (1–39 h/wk)	2.5 (1.1–5.6)
Full time (>40 h/wk)	10.7 (4.2–27.0)
Use of gloves during cattle birth care	
Fully compliant	0.4 (0.2–0.8)
Partly or noncompliant	Reference
No birth care	0.7 (0.4–1.1)
Contact with pigs at own or other farm	
Yes	2.4 (1.1–5.1)
No	Reference
Contact with poultry at own farm	
Yes	0.5 (0.3–0.8)
No	Reference
Indirect contact with rats/mice at own farm	
Yes	1.6 (1.0–2.7)
No	Reference
No. cows on farm in 2008†	1.0 (1.0–1.0)
Farm region	
South	1.9 (1.2–3.1)
Other	Reference
Automatic milking	
Yes	0.6 (0.4–1.0)
No	Reference
Birds in stable	
Yes	2.3 (1.2–4.4)
No	Reference
Contact with cows at other farm	
Yes	1.8 (1.0–3.2)
No	Reference
Contact with compost	
Yes	0.6 (0.3–0.9)
No	Reference
Beef cattle on the farm	
Yes	1.9 (1.0–3.7)
No	Reference

*The analysis included the primary participant- and farm-based characteristics associated with positivity (p<0.10 in likelihood ratio test). The number of observations was 708; the number of levels used was 309 (quasi-likelihood under the independence model criterion 695.52). OR, odds ratio. †Risk increases per cow.

## Discussion

The overall seroprevalence of 72.1% among DCF residents, including employees, was high, indicating a considerable lifetime risk for acquiring *C. burnetii* infection. Seroprevalence was highest among farmers (87.2%). The observed seroprevalence was similar to that determined by a study from the 1980s that showed an estimated seroprevalence of 68.0% among 94 Dutch dairy farm residents; however, laboratory methods used in that study were different than those used by us (*11*). The 72.1% seroprevalence was also compatible with recent estimates among dairy goat farms residents (68.7%) (*12*), dairy sheep farms residents (66.7%) (*13*), and livestock veterinarians (65.1%) (*14*). Estimates for these livestock-associated groups exceed the seroprevalence of 2.4% for the Dutch population during the pre-epidemic period, 2006–2007 (*2*), and the seroprevalences of 12.2% and 24.0% among persons residing in the most affected outbreak areas during the epidemic in the Netherlands (*15**,**16*).

Seroprevalence studies of other farmer populations, particularly dairy cattle farmers, are scarce, and, in general, it is difficult to compare international studies because of different study populations, tests, or cutoff values used. However, published seroprevalence estimates are generally lower than what we observed. A study using IFA with the same cutoff value that we used estimated a seroprevalence of 27.0% among a UK farm cohort (385 residents/workers) (*17*). Two other studies used a *C. burnetii* phase II IgG ELISA, which is somewhat less sensitive than IFA (*9*), and obtained seroprevalence estimates of 48.8% among Northern Ireland farmers from all types of farms (*18*) and 16.0% among 262 farm residents from 105 DCFs in Germany (*19*). A seroprevalence of 3.0% was observed in 163 residents from 100 farms (most likely cattle or pig) in Denmark; the study used the same IFA that we used, but cutoff values of IgG phase I and II were higher (≥512 and >1,024, respectively) (*20*). Using the same cutoff, we would obtain a comparable seroprevalence estimate of 2.7%.

Farm residents living in the southern part of the Netherlands were more likely to be seropositive. This was not surprising because living in the south was a risk factor for dairy goat farmers (*12*). In general, it is possible that seropositive DCF residents were partially affected by the many *C. burnetii*–positive BTM small-ruminant farms nearby. This possibility is supported by the close distance between residential addresses of persons who had a relatively recent infection and nearby *C. burnetii*–positive BTM small-ruminant farms. As determined on the basis of phase II IgM, 1.2% of DCF residents and 11.0% of small-ruminant dairy farm residents had a relatively recent *C. burnetii* infection (*12*,*13*), indicating that the infection among DCF residents was generally in the more distant past. Physicians diagnosed Q fever in 0.5% of DCF residents in our study compared with 4.1% in Dutch goat farm residents (*12*); nevertheless, to ensure a timely diagnosis and treatment, physicians should consider Q fever in patients with compatible symptoms and occupational exposure to cattle (*20*,*21*). In general, clinical illness from *C. burnetii* infection appears to be rare among DCF residents, which fits the suggestion in the literature that cattle-acquired *C. burnetii* infection has a milder clinical course (*20*). In other European countries and the United States, *C. burnetii* infection is endemic in cattle and in humans occupationally exposed to cattle, but there are few clinical cases of acute Q fever (*22*,*23*). A possible explanation is that abortion in late gestation is a key sign of infection in small dairy ruminants, but this is not the case in cattle. *C. burnetii* shedding by cattle is generally lower than that by small ruminants; concomitant and persistent shedding patterns are more frequent in clinically affected cows than healthy ones (*24*–*29*). Furthermore, sheep and goats have seasonal reproduction cycles and generally larger herd sizes, leading to huge amounts of bacteria shed during a short period. Multilocus variable-number tandem-repeat analysis genotyping has indicated that *C. burnetii* genotypes in dairy cattle herds and dairy consumer products (*30*,*31*), except for 1 placenta sample, are clearly distinct from the predominant outbreak genotype found at Dutch small ruminant dairy farms in 2007–2009 (*32*). Upcoming research should elucidate whether the cattle strains circulating in the Netherlands and other countries are less virulent.

Persons >55 years of age were at increased risk for seropositivity, which cannot be explained by differences in specific cattle-related tasks, frequency of cattle contact, or hours worked. It may be that host factors or continuous or regular exposure to the bacterium (booster effect) play a role that cannot adequately be assessed through a questionnaire. Full-time farm employment (>40 h/week) was a risk factor, which corresponds with a study among Dutch livestock veterinarians in which >30 hours of weekly animal contact was a risk factor for infection (*14*). Full-time farm employment and working or residing on a dairy (primarily) farm were risk factors in a UK farm cohort (*17*), indicating a dose-response relationship between seropositivity and the number of working hours spent with dairy cattle or in a dairy farm environment in general.

We identified several cattle-related risk factors for seropositivity among cattle farm residents/staff: herd size, cattle contact at other farms, and presence of beef cattle on their own farm. A larger herd size could pose a risk because of an increased chance for *C. burnetii* introduction or the presence of a larger susceptible population of cows; however, some farm-based risk factors associated with a large herd that were not assessed through the questionnaire might also have caused this effect (*19*,*33*,*34*). Cattle contact at other farms possibly reflects risk from exposure to *C. burnetii* in other infected herds. The presence of beef cattle as a risk factor for DCF residents is not easily explained, but it might reflect risk from more intense birth care and, therefore, more extensive human contact with cattle and birth products.

Protective factors included use of automatic milking and fully compliant use of gloves during birth care. Birth products of *C. burnetii*–infected ruminants are a source of human infections. A German study among veterinarians identified an association between increasing numbers of cattle obstetric procedures performed and seropositivity (*21*). Pig contact, indirect contact with rats/mice, and presence of wild or domesticated birds in the stable were indicated as risk factors in our study. Studies among veterinarians in the Netherlands and the United States identified swine contact as a risk factor (*14*,*35*); however, *C. burnetii* has not been found in pigs in the Netherlands (*30*). Rats and wild birds were identified as *C. burnetii* reservoirs in several studies (*36*–*38*) and as reservoirs on cattle farms in the Netherlands (*39*).

Fully compliant use of gloves during birth care can help farmers protect themselves against *C. burnetii* infection (*21*). Consistent use of farm boots and working clothes for professional visitors was a protective factor in our additional multilevel model. It might appear that the use of protective clothing by visitors will prevent *C. burnetii* transmission to the visitor rather than the farmer; however, providing gloves and farm clothes for visitors indicates a state of optimal awareness on the farm with regard to communicable diseases. In addition, automatic milking of cows might reflect less direct cattle exposure, especially through avoiding contact with the udders, raw milk, manure, and genital fluids, and thus might limit the chance of infection. Statistical analyses indicated lower risk for seropositivity among farm residents exposed to poultry and to compost. We have no biologically plausible explanation for this finding, and the statistical effect might have occurred by chance. Raw milk consumption was a risk factor for seropositivity in German dairy cattle farmers (*19*). Although consumption of raw milk was not an independent risk factor in our study, 21.8% of farm residents reported daily drinking of raw milk. *C. burnetii* exposure during nonautomatic milking could still implicate the risk of inhaling contaminated aerosols during pretreatment of the cow or during accidental raw milk ingestion.

The relatively low response rate of 10.4% in this study can be explained by a general lack of motivation or awareness among cattle farmers because Q fever was mainly considered a problem among small-ruminant dairy farms. A general fear of consequences resulting from possible control measures targeting the cattle sector comparable with implemented control measures for Q fever in the small-ruminant sector might also have played a role. Study results are, however, considered representative for the Dutch dairy cattle sector because participating and nonparticipating farms were generally comparable.

The overall *C. burnetii* seroprevalence of 72.1% among DCF residents is high. Multilevel analysis identified several plausible risk factors (e.g., employment on a farm, larger herd size, and cattle contact at other farms). A farm location in the southern region as risk factor suggests *C. burnetii* transmission from small-ruminant dairy farms to cattle farm residents living nearby. Use of automatic milking and fully compliant use of gloves during birth care are plausible protective factors, indicating less direct contact with cattle and, thus, a reduced chance of animal-to-human transmission. The dairy cattle sector must inform farmers about potential sources of infection. Biosecurity measures are warranted; for example, wild birds and vermin should be kept out of stables, and farmers/staff should be educated regarding the consistent use of PPE, such as wearing gloves during birth assistance and invasive procedures. Physicians should consider Q fever in the differential diagnosis for dairy cattle farmers with compatible symptoms. Future studies should more explicitly assess the clinical effect of acute and chronic Q fever in humans who live or work on DCFs.
